# A Canine *c-kit* Novel Mutation Isolated from a Gastrointestinal Stromal Tumor (GIST) Retains the Ability to Form Dimers but Lacks Autophosphorylation

**DOI:** 10.3390/ani15101444

**Published:** 2025-05-16

**Authors:** Kei Shimakawa, So Doge, Masaki Michishita, Eri Tanabe, Tsuyoshi Tajima, Masato Kobayashi, Makoto Bonkobara, Masami Watanabe, Kazuhiko Ochiai, Yoshikazu Tanaka

**Affiliations:** 1 Laboratory of Veterinary Hygiene, School of Veterinary Science, Nippon Veterinary and Life Science University, Tokyo 180-8602, Japanytanaka@nvlu.ac.jp (Y.T.); 2 Laboratory of Veterinary Pathology, School of Veterinary Science, Nippon Veterinary and Life Science University, Tokyo 180-8602, Japanmichishita@nvlu.ac.jp (M.M.); 3 Laboratory of Veterinary Pharmacology, School of Veterinary Science, Nippon Veterinary and Life Science University, Tokyo 180-8602, Japan; t-tajima@nvlu.ac.jp; 4 Laboratory of Veterinary Reproduction, School of Veterinary Science, Nippon Veterinary and Life Science University, Tokyo 180-8602, Japan; masato.k@nvlu.ac.jp; 5 Laboratory of Veterinary Clinical Pathology, School of Veterinary Science, Nippon Veterinary and Life Science University, Tokyo 180-8602, Japan; bonkobara@nvlu.ac.jp; 6 Laboratory of Urology, Graduate School of Medicine, Dentistry and Pharmaceutical Sciences, Okayama University, Okayama 700-8558, Japan; mwcorrespondence@gmail.com

**Keywords:** autophosphorylation, canine, *c-kit*, GIST, KIT, loss-of-function mutation

## Abstract

Gastrointestinal stromal tumors (GISTs) are mesenchymal tumors that develop in the gastrointestinal tract; KIT mutations are present in both canine and human GISTs. In this study, genomic DNA was extracted from formalin-fixed paraffin-embedded (FFPE) sections of 55 canine GIST cases, and mutation searches were performed for exons 8, 9, and 11. The results revealed novel mutations, A434T and F436S, in exon 8. In contrast to the A434T mutation without functional changes, the F436S mutant retained its dimerization ability, but lost its phosphorylation function and attenuated downstream Akt signaling, which is reflected in wound healing and migration activities. A comparison of the subcellular localization of WT KIT and the F436S mutant revealed no differences. In silico simulations indicated that the F436S mutation alters the structure of the near-membrane region and that its effects may extend to the transmembrane and intracellular domains compared to the WT. F436S is a point mutation that affects the entire molecule because co-mutation with the F436S mutation and the known autophosphorylation mutation reduces the autophosphorylation abilities.

## Simple Summary

Gastrointestinal stromal tumors are refractory tumors that affect both dogs and humans, and are partly caused by mutations in the *c-kit* gene. In this study, two novel mutations were identified in the *c-kit* gene in canine gastrointestinal tumor tissues: an alanine to threonine substitution at residue 434 and a phenylalanine to serine substitution at residue 436 (F436S). The F436S mutation is a loss-of-function mutation that does not aberrantly affect the intracellular molecular maturation mechanism but lacks the autophosphorylation function of KIT while maintaining the dimerization function required for the phosphorylation of downstream genes. Although gain-of-function mutations in canine KIT have been previously identified, this is the first report of a loss-of-function mutation.

## 1. Introduction

Gastrointestinal stromal tumors (GISTs) are common mesenchymal tumors that occur in the gastrointestinal tract of humans and dogs, and are derived from the interstitial cells of Cajal or their progenitor cells, which are pacemaker cells of the digestive tract [1]. Approximately 80% of human GISTs and canine GISTs have *c-kit* gene mutations [1,2]. KIT, the transcript of *c-kit* gene, is a type III receptor tyrosine kinase that comprises an extracellular domain composed of five immunoglobulin-like domains (IgDs), a transmembrane domain, a juxta membrane domain, and an intracellular domain composed of a kinase domain divided into an ATP-binding site and an activation loop by a kinase insertion sequence [3,4]. Among these four domains, the intracellular domain is important for regulating KIT kinase activity [4]. When stem cell factor (SCF), a ligand of KIT, binds to the extracellular domain, KIT forms a homodimer and the TK1 region is phosphorylated by ATP-derived phosphate in the cytosol. Phosphorylated KIT functions as a kinase and activates the downstream MAPK/ERK and PI3K/Akt pathways to promote cell survival, adhesion, migration, and differentiation [5,6]. The majority of *c-kit* mutations in human GISTs are found in exon 11 (approximately 70% of the total), followed by exon 9 (6% of the total), and less frequently in exons 8, 13, and 17 [1]. Exon 11 of KIT is a highly conserved juxta membrane region located in the transmembrane domain, and the reported mutations include missense mutations, deletions, and internal tandem duplications (ITDs) [7,8]. Surgical resection is the first-line treatment for human GIST, but treatment with imatinib mesylate, a tyrosine kinase inhibitor (TKI), is applicable for unresectable, metastatic, or recurrent GISTs with *c-kit* gene mutations [9]. Imatinib is a molecular-targeted drug that competitively inhibits autophosphorylation enhanced by structurally mutated KIT, suppressing excessive kinase activity [10]. However, some KIT-mutant GISTs have been reported to be resistant to TKI therapy; therefore, it is essential to identify the mutation type [11].

In dogs, *c-kit* mutations have been discovered in mast cell tumors and studied as applicable cases for TKI therapy [8,12]. Although TKI therapy has been used in cases of mast cell tumors with many *c-kit* mutations, some cases have been successful, whereas others have not, depending on the mutation site [13,14,15,16]. *C-kit* gene mutations have also been reported in canine GISTs, most of which are located in exon 11, as with humans [17,18,19,20]. TKI therapy has also been used to treat canine GISTs with some efficacy [21,22,23]. However, because TKI therapy for canine mast cell tumors does not result in a complete response in all cases, the response of GISTs to TKI therapy should be analyzed in the context of the KIT gene mutation profile [24].

In humans, *c-kit* mutations are thought to be involved in tumorigenesis isolated from tumors such as acute myeloid leukemia (AML), mastocytosis, and GISTs, and there are numerous reports of ligand-independent autophosphorylation gain-of-function mutations [25,26]. In addition, protein structural analysis of a previously reported mutant *c-kit*, which has a mutation within the dimer-forming region, showed that the mutation alters the conformation of the region around the mutation site, allowing ligand-independent dimer formation [27]. However, structural analyses of KIT mutations in dogs, which are also susceptible to GIST similar to humans [28], remain limited. Understanding these mechanisms is crucial for improving therapeutic strategies. This study aimed to identify novel mutations in c-kit exons 8, 9, and 11 in dogs diagnosed with GIST and analyze their effects on KIT function using biochemical, cellular, and structural biology approaches.

## 2. Materials and Methods

### 2.1. Histological Analysis

With permission from the University Ethics Committee, tissue samples were obtained from the Department of Veterinary Pathology, School of Veterinary Science, Nippon Veterinary and Life Science University (approval number 11-50, 27 May 2018). Two veterinary pathologists diagnosed all 55 cases by double-blind analysis according to the World Health Organization classification [29] (Table S1). Formalin-fixed paraffin-embedded (FFPE) GIST tissues were sliced at a thickness of 4 μm, sections were placed on slides, and hematoxylin and eosin (HE) staining was performed. Anti-DOG1 (1:500; SP31, Cell Marque, Rocklin, CA, USA) and KIT (1:1000; Dako, Glostrup, Denmark) antibodies were used for immunohistochemical staining according to standard protocols using dextran polymer reagent (anti-rabbit immunoglobulin EnVision + DAB system; Dako). For antigen retrieval, slides were autoclaved at 121 °C for 15 min in Tris-EDTA buffer (pH 9.0) or citrate buffer (pH 6.0). The slides were counterstained with hematoxylin. Normal rabbit IgG (Santa Cruz Biotechnology, Dallas, TX, USA) was used as a negative control. Images were acquired using a BX53 microscope (Olympus, Tokyo, Japan).

### 2.2. Sample Preparation and Sequencing

Genomic DNA from FFPE tissues from paraffin scrolls (Table S1) was extracted from canine GIST samples using the QIAamp DNA FFPE Tissue Kit (Qiagen, Hilden, Germany), according to the manufacturer’s instructions. The concentration and purity of the extracted DNA were determined by an ultraviolet absorption method, resulting in a range of 25–250 ng of DNA per sample. PCR amplification was performed using a Quick Taq HS dye mix (Toyobo, Osaka, Japan). The primer pairs used for amplifying the *c-kit* exons are listed in Table 1. Sequence data were directly determined using an ABI 3100-Avant Genetic Analyzer (Applied Biosystems, Foster City, CA, USA). For sequence analysis, canine *c-kit* (GenBank accession number: NP_001003181.1) was compared.

### 2.3. Cells and Cell Culture

AD-293 cells were purchased from Agilent Technologies (Santa Clara, CA, USA). and maintained in Dulbecco’s modified Eagle’s medium (DMEM; Wako, Osaka, Japan) supplemented with 10% fetal bovine serum, 100 U/mL penicillin, and 100 μg/mL streptomycin (Thermo Fisher Scientific, Waltham, MA, USA) at 37 °C in an atmosphere containing 5% CO_2_.

### 2.4. Generation of Hemagglutinin-Tagged KIT Mutants into the Mammalian Expression Vectors

Using a plasmid in which the wild-type *c-kit* gene was tagged with a C-terminal hemagglutinin (HA) epitope and cloned into a pcDNA3.1(+) vector (Clontech Laboratories, Inc., Palo Alto, CA, USA) [30], c.1300G>A (p.Ala434Thr: A434T) and c.1307T>C (p.Phe436Ser: P436S) mutants were constructed using overlap PCR mutagenesis. To construct the A434T and F436S mutants, nucleotide substitutions were performed by PCR mutagenesis using the WT sequence as the template and the following oligonucleotide primers: *c-kit*-A434T-F (5′-GTGTGGTTACAGGAT-TCCCAGAG-3′), *c-kit*-A434T-R (5′-CTCTGGGAATCCTGTAACCACAC-3′), *c-kit*-F436S-F (5′-GTGGTTGCAGGATCCCCAGAG-3′), and *c-kit*-F436S-R (5′-CTCTG-GGGATCCTGCAACCAC-3′). These mutants were amplified by PCR using the following primer sets: *c-kit*_F (5′-TTTAAACTTAAGCTTGGTACCTTGGGCGC-GAGCAGGAAC-3′) and *c-kit*_R (5′-TAGACTCGAGCGGCCGCTCAAGCGT-AATCTGGAACATC-3′) and cloned into pcDNA3.1(+), which was cut with *Hind*III and *Not*I (Clontech, Palo Alto, CA, USA) in the in-fusion cloning system (Takara Bio, Shiga, Japan).

### 2.5. Transfection and Adding SCF

AD-293 cells (5 × 10^5^) were seeded in 6-well plates and transfected with 2 µg of canine *c-kit* plasmid per well using FuGENE HD (Promega, Madison, WI, USA). Forty-eight hours after transfection, 100 ng/mL recombinant canine SCF: R&D Systems, Minneapolis, MN, USA) was added to the cell culture medium. Thirty minutes later, the cells were collected.

### 2.6. Western Blot Analysis

Cells were lysed with Mammalian Lysis Buffer (Promega) supplemented with a protease inhibitor cocktail (Promega). The insoluble fragments were removed by centrifugation at 16,000× *g* for 10 min at 4 °C and the supernatants were collected. The protein concentrations were determined using a Bicinchoninate Protein Assay kit (Nacalai Tesque, Kyoto, Japan). Approximately 5 μg of the extracted protein was analyzed by Western blot (WB) with the following antibodies: anti-KIT (1:2000; 18696-1-AP, Proteintech, Tokyo, Japan), anti-phospho-KIT (1:1000; 3073, Cell Signaling Technology, CST, Danvers, MA, USA), anti-Akt (1:1000; 4691, CST), rabbit polyclonal anti-p-Akt (1:1000; 4060, CST), anti-Halo (1:1000; G9281, Promega), and anti-HA (1:2000; 26183, Thermo, Waltham, MA, USA), anti-α-tubulin (1:2000; 013-25033, Wako, Kanagawa, Japan). Horseradish peroxidase-conjugated anti-mouse IgG (1:5000; 7076, CST) and anti-rabbit IgG (1:5000; 7074, CST) antibodies were used. EzWestLumi Plus (ATTO Corporation, Tokyo, Japan) was used to detect the antibody-bound proteins.

### 2.7. Cloning of c-kit-Expressing Cells

AD-293 cells without endogenous *c-kit* expression were transfected with various *c-kit*s cloned into pcDNA3.1 with the neomycin resistance gene and selected in a medium containing 400 μg/mL G418 (Thermo). The KIT expression status in each cell line selected using the G418-supplemented medium was confirmed by WB.

### 2.8. Cell Migration Assay with SCF Stimulation

The effect of *c-kit* mutants on cell migration was assessed using a wound-healing assay [31]. AD-293 cells stably expressing WT or mutant *c-kit* were seeded with 400 μg/mL G418 and grown in confluent monolayers in 6-well plates at a density of 4 × 10^5^ cells/well for 24 h. A single scratch was made using a sterile micropipette tip. Subsequently, the cell debris was removed by washing the plates twice with phosphate-buffered saline (PBS), and the cell culture medium was replaced with DMEM containing 0.5% FBS. The cells were then cultured for 72 h. Three independent experiments were performed and the area of cell migration was examined using an inverted microscope (DM IL LED; Leica Microsystems, Wetzlar, Germany) and quantified using ImageJ ver. 1.54e software (National Institutes of Health).

### 2.9. Immunostaining

Immunocytochemical staining for *c-kit* in AD-293 cells overexpressing KIT was performed by co-staining the Golgi body using the GOLGI IDTM Green Assay Kit (Enzo Life Sciences, Long Island, NY, USA). Cells were plated and cultured to 30–40% confluency in LabTek chambers (Nalgene, Rochester, NY, USA) and transfected with the pHTC-CMV/Neo vector (Promega) containing canine *c-kit* using the FuGENE HD Transfection Reagent (Promega). Forty-eight hours after transfection, the cells were fixed with 4% paraformaldehyde in 100 mM phosphate buffer and blocked with 5% normal goat serum in PBS. The cells were incubated with rabbit polyclonal anti-Halo antibodies (1:200 dilution; G9281, Promega) overnight at 4 °C and then with fluorescein isothiocyanate-conjugated anti-rabbit secondary antibody (Molecular Probes) for 2 h. To stain the nuclei, cells were incubated with Hoechst 33342 (Dojindo, Tokyo, Japan) for 15 min at 23 °C. Fluorescence staining was visualized under a fluorescence microscope (BZ-9000; Keyence, Osaka, Japan) and captured with BZ-II Analyzer Ver. 1.42 (Keyence).

### 2.10. Membrane Protein Extraction

Ten million cells per sample of AD-293 cells transfected with HA-tagged canine *c-kit* 48 h prior were collected and washed with cold PBS at 4 °C. Membrane proteins were extracted using a Trident Membrane Protein Extraction Kit (GeneTex, Irvine, CA, USA). The extracted proteins were detected by Western blot, and the purity of each fraction was assessed by Western blot for α-tubulin.

### 2.11. Halo-Tag Pull-Down Assay

We constructed a HA-tagged canine *c-kit* in the pcDNA3.1(+) vector (Clontech, Palo Alto, CA, USA) and canine *c-kit* in the pHTC-CMV/Neo vector (Promega). The expression of the Halo- and HA-tagged canine *c-kit* was induced in AD-293 cells using the FuGENE HD Transfection Reagent (Promega), and the transfected cells were grown for 36 h. After 12 h of serum starvation, cells were incubated for 30 min with 100 ng/mL SCF. The cells were harvested by centrifugation and washed with PBS. The cells were lysed in Mammalian Lysis Buffer (Promega) with a protease inhibitor cocktail for 15 min, and the cellular debris was cleared by centrifugation. In total, 100 µL of Magne Halo-Tag Beads (Promega, Madison, WI, USA) equilibrated with TBS containing 0.05% IGEPAL CA-630 (TBS+) was added to the supernatant. The samples were incubated overnight at 4 °C with rotation. The supernatant was discarded, and the beads were washed three times with TBS+ and suspended in SDS-PAGE loading buffer. The samples were analyzed using WB.

### 2.12. Structure Prediction of KIT F436S by Modeling

The crystal structure of the human KIT extramembrane region (PDB ID: 8dfm) was visualized using the University of California, San Francisco (UCSF) Chimera 1.17.3 software (http://www.cgl.ucsf.edu/chimera/, accessed on 15 May 2024) [32]. A431 and F433 of human KIT (corresponding to A434 and F436 of canine KIT, respectively) were mutated to Thr (T) and Ser (S), respectively, using the Dunbrack backbone-dependent rotamer library algorithm of the UCSF-Chimera software. The contacts between the residues at positions 431 and 433 in the chain were calculated and are shown for A431T and F433S, respectively. The solid blue lines signify stable contacts, as determined using the chimera program. Extrapolation of the extracellular domain of the canine KIT molecule to the human molecule was performed using SWISS-MODEL (https://swissmodel.expasy.org/, accessed on 16 May 2024) and the F436S mutant membrane proximity domain was visualized using PyMOL (https://pymol.org/, accessed on 16 May 2024).

### 2.13. Statistical Analysis

Data are expressed as the mean ± SD (SD). Analysis of variance (ANOVA) with Tukey’s post hoc test was performed when multiple comparisons were required. Significance was assessed at the 0.05 (or lower) level for all tests.

## 3. Results

### 3.1. Novel c-kit Mutations Have Also Been Detected in Canine GIST Tissues

**Figure 1:**
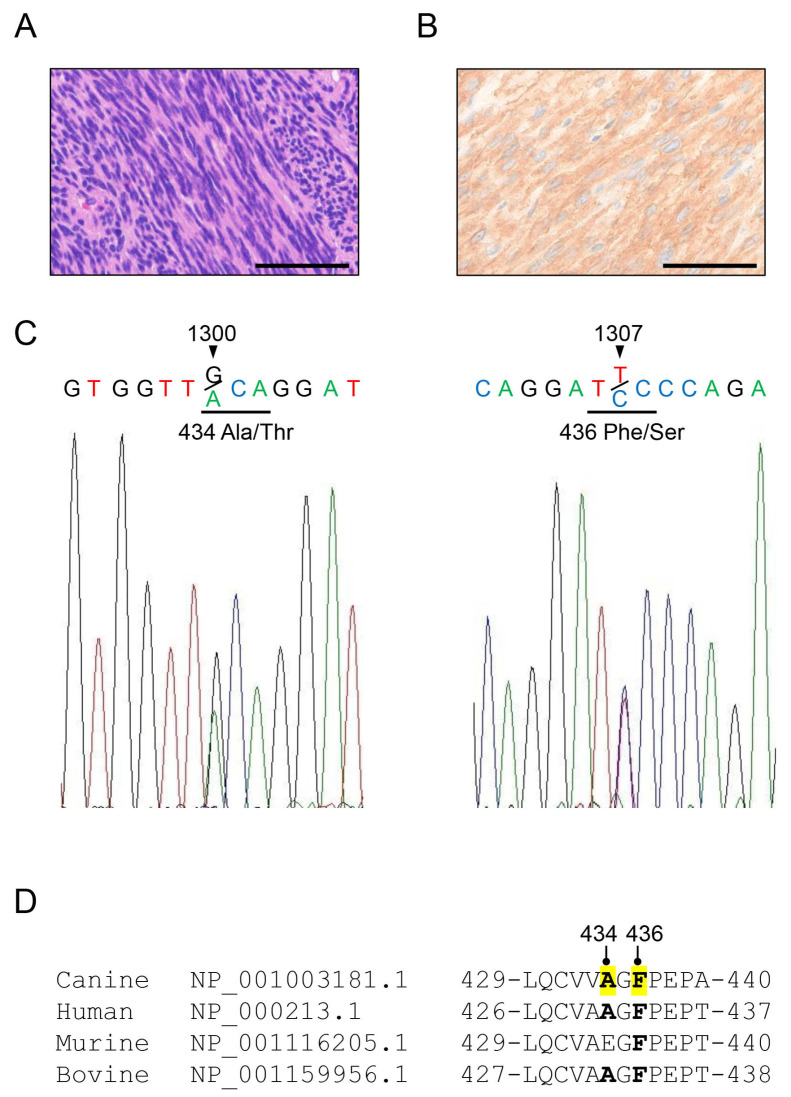
Histological findings of GIST and a novel mutation in canine c-kit were identified in the tumor tissue. (**A**) Hematoxylin–eosin (HE) staining of a representative case of GIST. Spindle cells showed a fascicular proliferative pattern. (**B**) Immunohistochemical staining of DOG1. Spindle cells were strongly positive for DOG1. Scale bars = 50 μm. (**C**) The electropherogram shows the nucleotide substitutions at positions c.1300G>A and c.1307T>C in the canine c-kit gene (GenBank accession: NM_001003181.1) amplified from genomic DNA of canine GIST tissues. The nucleotide sequences 1300–1302 and 1306–1308 encoding Ala/Thr 434 or Phe/Ser 436 are underlined. (**D**) Amino acid sequence alignments of canine, human murine, and bovine KIT proteins (GenBank accession numbers: NP_001003181.1, NP_000213.1, NP_001116205.1, and NP_001159956.1). Homologous residues 434 and 436 of the canine KIT are shown in bold.

In this study, 55 cases discovered to have GIST 1 (DOG1) or KIT positivity according to immunohistochemistry were evaluated as GISTs and were used in subsequent studies (Table S1). Figure 1A and B show representative GIST tissues stained with HE and DOG1 immunohistochemistry. Spindle cells showed a fascicular proliferative pattern (Figure 1A) and the cytoplasm was positive for DOG1 (Figure 1B). Canine c-kit exons 8, 9, and 11 were successfully PCR-amplified in 49 of the 55 FFPE tissue samples pathologically diagnosed as GISTs. Sequence analysis revealed two novel mutations, c.1300G>A (p.Ala434Thr: A434T) and c.1307T>C (p.Pro436Ser: P436S), as well as known polymorphisms and mutations in exons 8 and 11 (Table S2; Figure 1C). Comparison of the novel mutation neighborhood regions of canine c-kit exon 8 protein (GenBank accession: NP_001003181.1) with human, murine, and bovine c-kit proteins (NP_000213.1, NP_001116205.1, and NP_001159956.1). Sequence alignment showed that canine Ala434 and Phe436 corresponded to human Ala431 and Phe433, respectively, and this sequence is highly conserved in other species (Figure 1D).

### 3.2. The Canine KIT Mutant of F433S Lacks Autophosphorylation Ability upon SCF Addition

**Figure 2:**
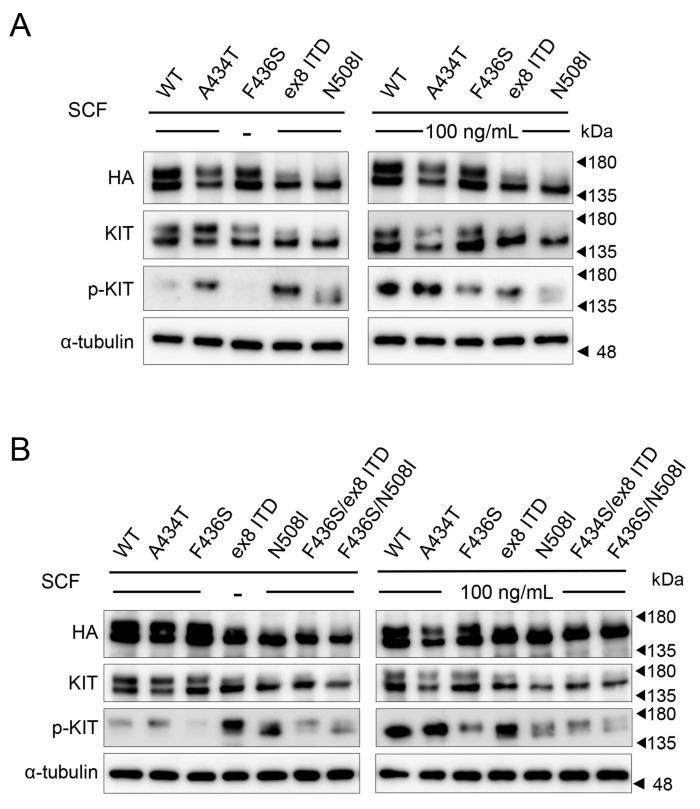
Phosphorylation profiles of KIT mutants with or without SCF stimulation. (**A**) AD-293 cells were overexpressing various HA-tagged canine KIT proteins, and the phosphorylation status of KIT in the presence and absence of SCF was observed by Western blot. α-tubulin was used as a loading control. (**B**) The autophosphorylation ability of co-mutants of F436S mutated KIT and known gain-of-function mutations (ex8 ITD or N508I) was analyzed by Western blot.

To investigate the autophosphorylation profile of novel canine KIT mutants with and without SCF, we used AD-293 cells lacking KIT expression [33]. Overexpression of WT, A434T, or F436S mutants of KIT and Western blot analysis showed that autophosphorylation was considerably attenuated in A436S mutant-expressing cells in the presence and absence of SCF, compared to WT and other mutants (Figure 2A). To analyze the suppression of autophosphorylation by the F436S mutation, we generated a co-mutant with gain-of-autophosphorylation mutations such as KIT exon 8 ITD or N508I [28,34]. The F436S/ex8 ITD and F436S/N508I mutants showed attenuated phosphorylation with and without SCF stimulation compared to the single ex8 ITD or N508I mutants (Figure 2B).

### 3.3. Suppression of Cell Proliferation and Migration by KIT F436S Mutant

**Figure 3:**
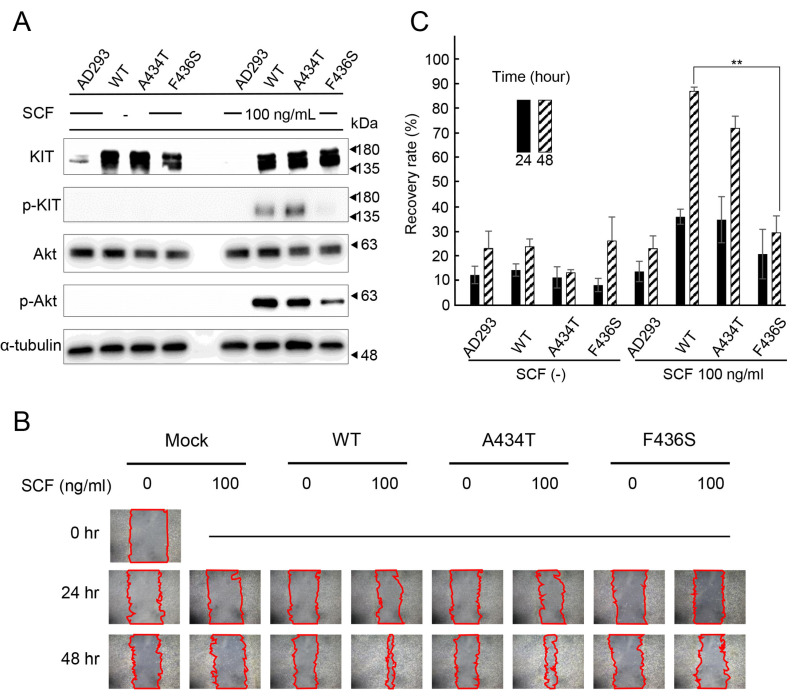
Migration activity of AD-293 cells stably expressing various canine KIT mutants (**A**) The phosphorylation status of p-kit and p-Akt was assessed by Western blot in AD-293 cells stably expressing KIT WT, A434T, or F436S mutants. (**B**) The migration ability of AD-293 cell lines stably expressing WT, A434T, or F436S mutant of canine KIT was assessed by a wound-healing assay after 0, 24, and 48 h of scratching. Representative images of scratched areas are shown. Microscopic magnification ×200. **(C)** Cell migration was quantified using ImageJ software. The recovery rate was quantified as 100 of the cell growth area before scratching, and the values are shown as the mean ± SD from three independent experiments. Asterisks on top of brackets indicate significant differences calculated by ANOVA with Tukey’s multiple comparison test (** *p* < 0.01).

To investigate the effect of KIT on cells, we generated stable cell lines expressing the WT, A434T, or F436S mutants. Compared to the WT or A434T mutant cells, F436S mutant cells showed attenuated Akt phosphorylation in the presence of SCF, as determined by Western blot analysis (Figure 3A). F436S-expressing cells also showed a significantly reduced recovery rate compared with WT and A434T-expressing cells 48 h after scratching with SCF (Figure 3B,C).

### 3.4. The F436S Mutation Did Not Affect Protein Maturation or Subcellular Localization of the Canine KIT

**Figure 4:**
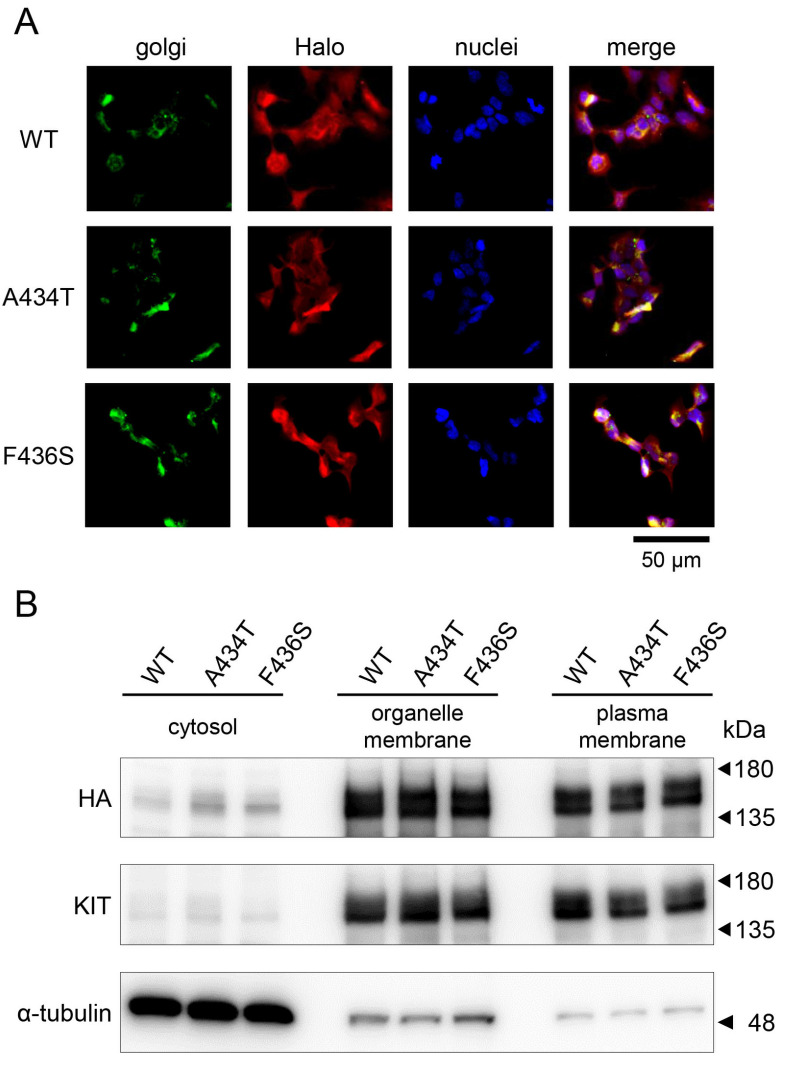
Intracellular dynamics of KIT mutants. (**A**) Subcellular localization of various Halo-tagged KITs (red) forcibly expressed in AD-293 cells determined by co-staining with the Golgi apparatus (green). Representative cell images expressing each KIT are shown with merged colocalization with the cell nuclei (blue). (**B**) Cellular proteins were fractionated into cytosol, organelle membrane, and plasma membrane fractions, and the localization of KIT was analyzed by Western blot. α-tubulin was used as a loading control.

To examine the subcellular localization of F436S mutant KIT, immunostaining for overexpression of Halo-tagged KIT in the Golgi apparatus revealed no differences in localization among the WT, A434T, and F436S mutants (Figure 4A). Cytosols, organelles, or plasma membranes were isolated from cells and analyzed for KIT expression patterns by WB, and no differences were found between WT- and mutant-expressing cells (Figure 4B).

### 3.5. Loss of F433S Phosphorylation of KIT Did Not Affect Its Ability to Form Dimers

**Figure 5:**
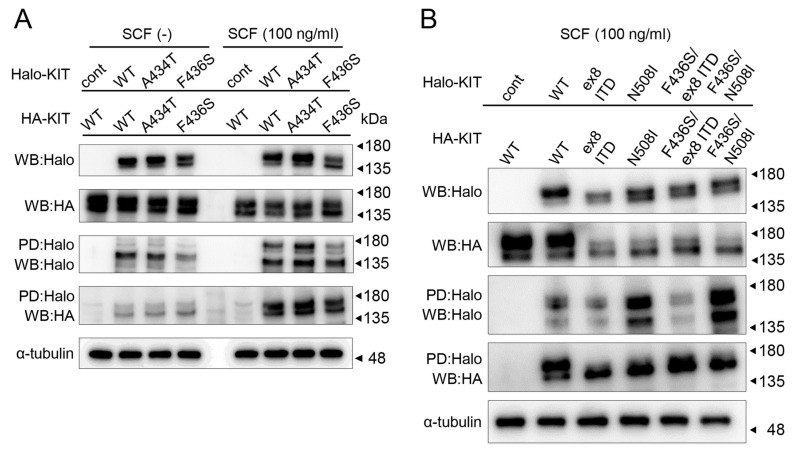
Evaluation of dimerization ability of canine KIT mutants. (**A**) For the Halo-Tag pull-down (PD) assay, Halo and HA-tagged KIT were co-transfected in AD-293 cells. Lysates from Halo-tagged REIC/DKK-3- and/or HA-tagged SGTA-transfected AD-293 cells were analyzed. The sample was pulled down using a Halo-tagged KIT and analyzed by Western blot (WB) using an anti-HA antibody. (**B**) Analysis of the dimer formation ability of co-mutants of F436S and known gain-of-function mutations (ex8 ITD or N508I) by PD assay.

Because the 433Phe residue of canine KIT is located in a region thought to be involved in the dimerization domain, we evaluated F436S’s ability to achieve dimerization; this was assessed via a Halo-Tag pull-down assay. The pull-down (PD) assay was performed using lysates of AD-293 cells expressing HA and Halo-tagged KIT following SCF stimulation. When Halo-tagged KIT was pulled down and WB was performed with an anti-HA antibody, no difference was found between the WT and mutants with or without SCF (Figure 5A). In addition, PD assays performed using the co-mutant of F436S and ex8 ITD/N508I, which forms dimers and auto-phosphorylates in the absence of SCF, showed no change in their ability to form dimers (Figure 5B).

### 3.6. Effects of F436S Mutation on the Conformation of Canine KIT

**Figure 6:**
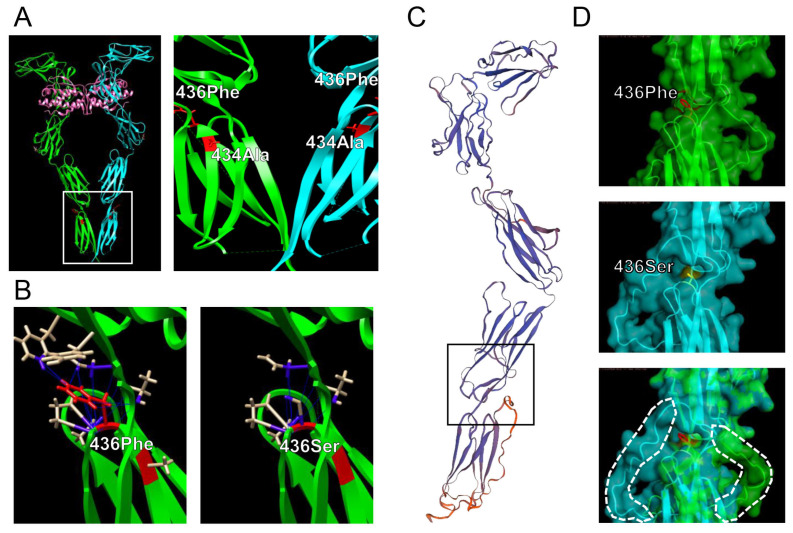
Effects of A434T and F436S mutations in canine KIT on the conformation of KIT. (**A**) Left panel: crystal structure of the human KIT extramembrane region (PDB ID: 8dfm). Right panel: enlargement of the white frame area in the left panel. The A434 and F436 residues were located near the D4 and D5 domains. (**B**) Left panel: The red-colored phenylalanine has 30 contacts with neighboring amino acids. Right panel: A decrease in the number of contacts (15 contacts) when residue 436 is substituted with Ser. (**C**) A canine KIT N-term molecular simulation model built using SWISS-MODEL based on PDB ID: 8dfm. (**D**) Upper panel: the molecular structure of the surrounding residue 436Phe canine KIT was visualized using the PyMOL program. Middle panel: 3D structure of KIT with residue 436 replaced with Ser using PyMOL program. Lower panel: Merged images of 436F and 436S. The white dotted-line area represents the molecular conformation that differs between 436F and 436S.

To verify the results of the functional analysis of KIT mutations, the protein structure editing tool in the UCSF-Chimera software was used to analyze the possible structural outcomes of F436S substitutions. The 433rd residue (corresponding to residue 436 of canine KIT) is located on the C-terminal side of human KIT (PDB ID: 8dfm) (Figure 6A). The UCSF-Chimera rotamer tool allows the simulation of amino acid side-chain substitutions [35]. The best rotamer for Ser at 436 residue was selected based on its side-chain torsion, the probability values in the rotamer library, and the context of the structural environment. Direct interactions (contacts/clashes) between residue 436 and surrounding amino acids were analyzed. The number of contacts decreased from 30 to 15 and no clashes appeared because of the amino acid substitution from Phe to Ser (Figure 6B). Residue 436 is located in the black square of the KIT extracellular domain extrapolated from canine KIT to PDB ID: 8dfm using the SWISS-MODEL program (Figure 6C). When 436Phe and 436Ser were drawn using the PyMOL program (PyMOL molecular graphics system, version 1.2r3pre, Schrödinger, LLC., New York, NY, USA) and merged, conformational changes were observed in the structure, which helped to form the C-terminus (Figure 6D).

## 4. Discussion

In this study, *c-kit* gene amplification was observed in 49 of the 55 patients diagnosed with GIST, and *c-kit* gene polymorphisms or mutations were detected in 30 patients (61%). The most common *c-kit* mutation was c.1710–1715dup, located near the plasma membrane in exon 11 (11/49 cases). In contrast, exon 8 had several silent polymorphisms, c.1275G>A, in 21/49 cases. Consistent with previous reports [2,18,20] that found a high percentage of *c-kit* exon 11 mutations in canine GIST tissues, our mutation analysis yielded similar results. In contrast, there are only a few exon 8 mutations in *c-kit* in canine GISTs, and the A434T and F436S mutations identified in this study are novel. Overexpression experiments revealed that the F436S mutant KIT lost its autophosphorylation ability upon the addition of SCF. Although several antibodies that detect KIT autophosphorylation have been reported [36,37], Y703, which was used in this study, is the only antibody that can detect canine KIT phosphorylation. Therefore, it is insufficient to study the autophosphorylation profile of KIT. However, the loss of KIT autophosphorylation ability due to a single amino acid mutation, found in this study, is unprecedented. Whether this mutation is the direct cause of GIST is unknown; however, it is unique as a KIT structural mutation that occurs in vivo. Furthermore, co-mutations of F436S with autophosphorylation-enhancing mutations, such as ex8 ITD and N508I, counteracted autophosphorylation. The results showed that the F436S mutation in canine KIT is a single amino acid substitution, but an important mutation that results in functional changes throughout the molecule. In addition, the possibility of the presence of F436S co-mutations in tyrosine kinase inhibitor-eligible cases, such as ex 8 ITD and N508I mutations, raises the possibility that the pathoetiology is not due to an enhanced KIT autophosphorylation capacity. Therefore, current canine KIT mutation analysis may not provide a basis for drug selection. It has also been reported that Cajal interneuron-mediated *c-kit*-deficient mice have decreased contraction of the gastrointestinal tract, resulting in intestinal inflammation [38]. Therefore, when the F436S mutation causes loss of its autophosphorylation ability, which is essential for the functional expression of KIT, it can lead to pathological conditions other than GIST, such as intestinal motility abnormalities. Because it has been reported that ligand-independent autophosphorylation gain mutant KIT is overexpressed in GIST, the expression status of the KIT loss of phosphorylation mutant identified in this study should also be examined in clinical cases [39].

Next, to investigate the effects of the loss of autophosphorylation mutation, we performed validation using cloned WT or mutant KIT-expressing AD-293 cells and found that the KIT F436S mutant clone showed decreased phosphorylation of Akt, which is downstream of KIT signaling, and decreased cell migration. Thus, the F436S mutation also affects the cellular level and reduces downstream signaling. While observing attenuated Akt phosphorylation in canine GIST tissues with the F436S mutation would have provided stronger evidence, available p-Akt antibodies did not show cross-reactivity in canine immunohistochemistry. Tumors expressing the KIT F436S mutant form may have reduced migration, resulting in slower tumor metastasis and invasion than those expressing the gain-functioning mutant of KIT. If KIT mutations alter the rate of tumor progression in vivo, the F436S mutation could also be used as a prognostic factor. Because one cause of the loss of function of KIT mutants is that they remain in the Golgi apparatus due to conformational changes and do not correctly localize to the plasma membrane [40], the subcellular localization of the F436S mutant was examined. We found no difference in subcellular localization between the WT and F436S mutants. Furthermore, there was no difference in the amount of protein in the cytoplasm, organelle membrane, or plasma membrane, indicating that the F436S mutant had no abnormal subcellular localization during molecular maturation.

Normally, KIT forms homodimers and exerts tyrosine kinase activity upon SCF binding. The F436S mutation, which causes a loss of autophosphorylation ability, is located in the membrane-proximal domain D5 (D5), which is involved in dimer formation [27,41]. Therefore, the F436S mutant was thought to have impaired dimer formation, a step before autophosphorylation; however, the results of the PD assays showed that it retained the ability to form dimers. Ligand-independent auto-phosphorylating KIT mutations do not require SCF binding for dimer formation [28]. Such mutant KIT allows ligand-independent dimer formation through a conformational change at the mutation site located in the D5 region [27]. Although F436S is a mutation in the dimer-forming region, it does not alter the dimer-forming ability and causes a loss of phosphorylation. This type of mutation has not been previously identified and adds new insights into the relationship between the dimer-forming ability and the phosphorylation ability of the KIT.

Because the F436S mutant retained its dimer-forming ability, we hypothesized that the loss of phosphorylation function in the F436S mutant was due to a cause other than the dysfunction of the dimer-forming domain. To investigate this hypothesis, we performed in silico analysis to predict the protein conformational change in F436S using a canine KIT. Using the UCSF-Chimera program, which is suitable for local observation of molecular structural changes, the number of contacts with amino acids around the D5 region was reduced when amino acid substitutions from phenol to serine were simulated. We considered the cause of this phenomenon to be that the benzyl group on the side chain of phenylalanine has a high molecular weight and numerous points of contact with the surrounding amino acids, whereas the hydroxymethyl group, which is smaller, has fewer points of contact. To analyze the effect of the F436S mutation on the 3D structure of the canine KIT protein molecule, a virtual canine KIT molecule was constructed using the SWISS-MODEL program [42,43]. Although we should have constructed a model of the entire canine KIT molecule, the structure of the entire length of the KIT molecule, which is the material used for model construction, was not registered in the database; therefore, we constructed the model from the N-terminus to D5 near the membrane region. The model was visualized using PyMOL 3.1 molecular graphics system software and compared to canine KIT WT and the F436S mutant, which showed a change in the orientation of the α-helix structure toward the transmembrane region [41]. Because the transmembrane domain structure leads to the KIT autophosphorylation region in the cell, changes in this region could affect the dimeric structure and hinder phosphate binding. However, structural analysis of the full-length canine KIT remains necessary. Further in vitro studies are also warranted to analyze the differences in KIT phosphorylation between the WT and F436S mutants.

## 5. Conclusions

Among the novel *c-kit* mutations identified in canine GIST cases, the F436S mutation retained dimer formation ability, but lost autophosphorylation and downstream Akt phosphorylation function in the presence of SCF. This mutation reduces cell proliferation and invasiveness, and co-mutation with a gain-of-function mutation was reported to decrease autophosphorylation function. In silico analysis indicated that the F436 residue, when mutated to serine, is located in the D5 region of KIT near the cell membrane and may induce structural changes in the adjacent intracellular phosphorylation region.

## Figures and Tables

**Table 1:** Primer sequences for canine *c-kit* amplification from GIST genomic DNA.

Purpose	Base Sequence	
	Forward	Reverse
exon 8	5′-gtcctcttcaaactcaagaagg-3′	5′-gtagccaaaataatcctctc-3′
exon 9	5′-gatggaatggacttaaaatcatg-3′	5′-gatggaatggacttaaaatcatg-3′
exon 11del	5′-catttgttctctaccctaagtgct-3′	5′-gtttccattgatctcctcaac-3′
exon 11ins	5′-cccatgtatgaagtacagtggaag-3′	5′-gttccctaaagtcattgttacacg-3′

## Data Availability

The data that support the findings of this study are available from the corresponding author upon reasonable request.

## Supplementary Materials

The following supporting information can be downloaded at https://www.mdpi.com/article/10.3390/ani15101444/s1, Table S1: DOG1 and KIT immunostaining of GIST tissues (n = 55); Table S2: Profiles of canine GIST cases in stromal tumors (n = 55).
