# Supplementary material for: A Canine c-kit Novel Mutation Isolated from a Gastrointestinal Stromal Tumor (GIST) Retains the Ability to Form Dimers but Lacks Autophosphorylation
Source: Animals (Basel). 2025 May 16;15(10):1444. doi: 10.3390/ani15101444 (PMC12108377; doi:10.3390/ani15101444)
Supplement: Supplementary file 1 [file animals-15-01444-s001.zip › Table_S1.pdf]

Table S1. DOG1 and c-kit immunostaining of GIST tissues (n = 55)

| Case | immunohistochemistry | | | |
|---|---|---|---|---|
| | DOG1 | | c-kit | |
| | Cytosol | Cell membrane | cytosol | Cell membrane |
| 1 | + | + + + | + | - |
| 2 | + + | + + + | - | - |
| 3 | + + | + | + + | + |
| 4 | + | + + | - | - |
| 5 | + | + + | + | + |
| 6 | + | + + | + + | + + |
| 7 | + | + + | - | - |
| 8 | + | + + | + | + + |
| 9 | + + | + + | + + | + |
| 10 | + | + + + | + | + + |
| 11 | + + | + + + | + + + | + + |
| 12 | + | + + + | + | + + + |
| 13 | + | + + | + + | + |
| 14 | + | + | - | - |
| 15 | + + + | + + + | + + | + + |
| 16 | + + + | + + | + | + |
| 17 | + + | + + + | + + | + |
| 18 | + + | + + | + + | + + + |
| 19 | + + | + + + | + + | + + |
| 20 | + | + | - | - |
| 21 | + | + + + | + + | + + + |
| 22 | + + | + + + | + + | + + |
| 23 | + + | + + | + + | + + |
| 24 | + + | + + + | + + | - |
| 25 | + + | + + + | + | + + |
| 26 | + | + + + | - | - |
| 27 | + + | + + | + | - |
| 28 | + + + | + + + | + + | + |
| 29 | + + | + | + + | - |
| 30 | + | + + | + + | + |
| 31 | + + | + + | + + | + |
| 32 | + | + + + | + + | + + |
| 33 | + + | + + + | ++ | + + |
| 34 | + | + + | + + | + + + |
| 35 | + + | + + | + + | + + + |
| 36 | + + | + + | + + | + + |
| 37 | + + | + + | + + | + |
| 38 | + + | + | + + | + |
| 39 | + | + + + | + | + |
| 40 | + + + | + + + | + + | + |
| 41 | + | + + | + | + |
| 42 | + | + | - | - |
| 43 | + + + | + + + | + + | + + |
| 44 | + + | + + | + | + + |
| 45 | + + | + + | + | + |
| 46 | + + | + + + | + + | + |
| 47 | + + | + + | - | - |
| 48 | + + | + + | ± | ± |
| 49 | + | + + | + | + |
| 50 | + + + | + + + | + + | + |
| 51 | + + | + | + + | + |
| 52 | + + + | + + + | + + | + |
| 53 | + + | + + | + | + |
| 54 | + | + + | + | + + |
| 55 | + + + | + + + | + + | + |

-, no staining; ±, partially staining (30-50%); +, weak staining; + +, moderate staining; + + +, strong staining
