# Supplementary material for: A Canine c-kit Novel Mutation Isolated from a Gastrointestinal Stromal Tumor (GIST) Retains the Ability to Form Dimers but Lacks Autophosphorylation
Source: Animals (Basel). 2025 May 16;15(10):1444. doi: 10.3390/ani15101444 (PMC12108377; doi:10.3390/ani15101444)
Supplement: Supplementary file 1 [file animals-15-01444-s001.zip › Table_S2.pdf]

Table S2. Profiles of canine GIST cases in stromal tumors (n = 55)

Case	Dog breed	Age	Gender	Site of occurrence	Known mutation	Novel mutation
1	Golden Retriever	11	F	Unknown	c.1710-1715dup	-
2	Miniature Dachshund	12	F	Unknown	NA	NA
3	Unknown	16	M	Unknown	c.1275G>A	-
4	Papillon	11	M	Unknown	-	-
5	Miniature Pinscher	14	F	Stomach	-	c.1300G>A
6	Welsh Corgi	12	M	Cecum	-	c.1307T>C
7	Miniature Dachshund	11	F	Small intestine	-	-
8	Miniature Dachshund	13	M	Cecum	c.1275G>A<br>c.1667-1672del	-
9	Welsh Corgi	14	F	Cecum	-	-
10	Miniature Dachshund	14	M	Small intestine	-	-
11	Miniature Dachshund	10	M	Small intestine	-	-
12	Mixed	13	M	Cecum	-	-
13	Miniature Dachshund	13	M	Stomach	c.1275G>A<br>c.1673-1675del	-
14	Labrador Retriever	12	M	Cecum	c.1275G>A<br>c.1710-1715dup	-
15	English Setter	8	M	Cecum	NA	NA
16	Toy Poodle	10	M	Unknown	c.1710-1715dup	-
17	Brittany Spaniel	11	M	Cecum	c.1275G>A<br>c.1710-1715dup	-
18	Welsh Corgi	13	F	Cecum	c.1710-1715dup	-
19	Miniature Schnauzer	14	M	Cecum	c.1275G>A<br>c.1710-1715dup	-
20	Welsh Corgi	10	M	Unknown	-	-
21	Miniature Dachshund	14	F	Cecum	c.1275G>A<br>c.1724T>C	-
22	Mixed	14	F	Cecum	c.1275G>A<br>c.1710-1715dup	-
23	Jack Russell Terrier	12	F	Stomach	c.1710-1715dup	-
24	Kishu Inu	10	M	Small intestine	c.1275G>A	-
25	Miniature Dachshund	15	F	Cecum	NA	NA
26	Boston Terrier	11	M	Unknown	c.1710-1715dup	-
27	Shiba Inu	9	F	Small intestine	NA	NA
28	Shih Tzu	15	F	Small intestine	c.1710-1715dup	-
29	Miniature Dachshund	11	M	Cecum	c.1275G>A	-
30	Pug	15	M	Small intestine	c.1275G>A<br>c.1710-1715dup	-
31	Cavalier King Charles Spaniel	12	M	Small intestine	-	-
32	Golden Retriever	9	F	Unknown	-	-
33	Miniature Schnauzer	13	M	Unknown	c.1275G>A	-
34	Pomeranian	11	F	Stomach	NA	NA
35	Mixed	15	M	Cecum	c.1287C>T<br>c.1724T>C	-
36	French Bulldog	10	F	Cecum	c.1275G>A	-
37	Toy Poodle	11	F	Small intestine	-	-
38	Mixed	11	F	Cecum	-	-
39	Mixed	12	F	Cecum	c.1275G>A	-
40	Mixed	12	F	Small intestine	-	-
41	English Setter	15	M	Cecum	-	-
42	Mixed	11	F	Unknown	-	-
43	Mixed	11	M	Cecum	-	-

Table S2. Profiles of canine GIST cases in stromal tumors (n = 55)

Case	Dog Breed	Age	Gender	Histology	Known mutation	Novel mutation
44	Shih Tzu	13	F	Small intestine	c.1275G>A<br>c.1673-1675del	-
45	Unknown	11	M	Small intestine	c.1275G>A	-
46	Golden Retriever	11	M	Cecum	-	-
47	Unknown	13	F	Stomach	NA	NA
48	American Cocker Spaniel	12	F	Small intestine	c.1275G>A	-
49	Mixed	13	F	Cecum	-	-
50	Shetland Sheepdog	11	F	Small intestine	-	-
51	Beagle	8	M	Cecum	c.1275G>A	-
52	Border Collie	14	M	Cecum	-	-
53	French Bulldog	12	M	Small intestine	c.1275G>A	-
54	Miniature Dachshund	15	M	Stomach	c.1275G>A	-
55	French Bulldog	12	F	Small intestine	c.1275G>A<br>c.1664-1669del	-
